# Modularity, homology, heterochrony: Gavin de Beer's legacy to the mammalian skull

**DOI:** 10.1098/rstb.2022.0078

**Published:** 2023-07-03

**Authors:** Brian K. Hall, James Hanken

**Affiliations:** ^1^ Department of Biology, Dalhousie University, Halifax, Nova Scotia, Canada B3H 4J1; ^2^ Museum of Comparative Zoology, Harvard University, Cambridge, MA 02138, USA

**Keywords:** Gavin de Beer, homology, heterochrony, modularity, mammalian skulls, segmentation

## Abstract

Modularity (segmentation), homology and heterochrony were essential concepts embraced by Gavin de Beer in his studies of the development and evolution of the vertebrate skull. While his pioneering contributions have stood the test of time, our understanding of the biological processes that underlie each concept has evolved. We assess de Beer's initial training as an experimental embryologist; his switch to comparative and descriptive studies of skulls, jaws and middle ear ossicles; and his later research on the mammalian skull, including his approach to head segmentation. The role of cells of neural crest and mesodermal origin in skull development, and developmental, palaeontological and molecular evidence for the origin of middle ear ossicles in the evolutionary transition from reptiles to mammals are used to illustrate our current understanding of modularity, homology and heterochrony.

This article is part of the theme issue ‘The mammalian skull: development, structure and function’.

## 1. Introduction

The phrase ‘the mammalian skull', whether intentionally or coincidentally, implies that the skulls of all mammals conform to an identical structural pattern that is evolutionarily conserved and distinct from that of all other vertebrates. But identifying a skull as mammalian may not be so straightforward. The best way to appreciate variation in skull morphology within and among living vertebrates,^1^ which may include shape, size, proportions and specific adaptations, is to visit a natural history museum, either in person or through an online search of their collections. If that is not an option, there is another. You could examine the beautiful photographs of over 300 skulls of more than 2000 vertebrates amassed by Alan Dudley—'one of the largest and most comprehensive [collections] in private hands anywhere in the world' [1, p. 14]. There you would see a commonality in skulls but also variation. The variation, however, lies within bounds that we recognize as mammalian or reptilian or avian, and which enclose what the palaeontologist David Raup [2] termed ‘morphospace'—the multidimensional range of shape or structure of a morphological character—although the concept goes back to D'Arcy Thomson and even earlier [3].

Gavin de Beer was acutely aware of this variation, and he devoted much of his professional life to unravelling the development and evolution of skulls. Before de Beer, skulls had been regarded as modified vertebrae; similarities were described between cranial bones and specific anterior vertebrae, as summarized in [4] in T. H. Huxley's Croonian Lecture ‘On the theory of the vertebrate skull’. Nowadays, the term skull is widely used just for the cranium, that mass of cartilage and bone that protects the brain and sense organs (eyes, ears and nose) and gives structure to the face. However, studies of ‘skulls' writ large also include the mandibular and hyoid arch skeletons and upper and lower jaws, as well as middle ear ossicles, all of which occupied de Beer's attention.

A primary concern then and now is what today is referred to as *modularity* of the skull and its component parts. As independent but interacting units (see below), modules are one of the levels of developmental integration that maintain the skull within the broad limits that we recognize as taxon-specific. The development of landmark-based geometric morphometrics has enormously enhanced our ability to identify such modules. A recent study on 13 species of ant-eating mammals, for example, identified six or seven functional modules and concluded that ‘despite some integration shifts related to extreme functional and morphological features of myrmecophagous skulls, cranial modular architectures have conserved the typical mammalian scheme' ([5, p. 1], and see references therein and [6]).

Modules can be recognized, named and classified at different levels—genes, cells, tissues, organs, regions of the body, functional units etc. As impressively articulated by de Beer [7] in *Homology: an unsolved problem*, we also recognize that biological components such as the skull may be homologous at one level but not at another. For example, the same cartilages or bones (or other skeletal elements that we recognize by the same name) in different species may be regarded as homologous even though they are derived from cells of different embryonic origin (§5).

As a third major contribution relating development to evolution, de Beer invoked the concept *of heterochrony*—change in the timing of development in a descendant compared with an ancestor—to ‘explain' the evolution of elements of the skull, jaws and middle ear ossicles (§3). In this paper, we explore de Beer's contributions to modularity, homology and heterochrony in the light of more recent research.

## 2. Gavin Rylands de Beer (1889–1972)

### (a) Early life

Born in Malden in the county of Surrey, England, in 1889, Gavin Rylands de Beer lived and was educated in France until he went to Harrow, an independent boarding school for boys in London. From there, he entered Magdalen College, Oxford for a term (1917) before joining the Grenadier Guards, an infantry regiment of the British Army, to fight in World War I. He returned to Magdalen in 1919, where he graduated in Zoology in 1922. A year later, de Beer was appointed a Fellow of Merton College and began teaching zoology at Oxford. In 1938, he moved to University College London (UCL) as Reader in Embryology. Service in the Grenadier Guards in the Second World War was followed by his appointment as Professor of Zoology at UCL (1945), and then as director of the British Museum (Natural History) for 10 years (1950–1960) until his retirement.^2^

A member of a family whose enormous wealth derived from their monopoly of diamond mining in South Africa, we obtain a glimpse of de Beer *the man* from Richard Fortey's brilliant history of the Natural History Museum. de Beer was ‘multi-lingual, a polyglot polymath … He was most extraordinarily clever, and very aware of the fact. He had ‘a pompous grandeur’ [and was] ‘vainglorious’ [10, pp. 263, 264, 265]. Short in physical stature, ‘he arrived and left the museum every day in his Rolls-Royce, immaculately besuited; it was common knowledge that he had to perch atop a pile of cushions to get a fair view of where he was going’ [10, p. 263]. Suits and ties were required for staff^3^ every day except Friday, when sports jackets with leather elbow patches were allowed in preparation for a weekend in the country [10, pp. 263–265]. de Beer's connections through marriage facilitated such a lifestyle; in 1925, he married Cicely Glyn Medylcott (1892–1973), fourth child of Sir Hubert James Medlycott, 6th Baronet. de Beer was elected Fellow of the Royal Society of London (FRS) in 1940, knighted in 1952, and received the Darwin Medal of the Royal Society in 1957 and the Kalinga Prize from UNESCO in 1968.

### (b) Embryologist and evolutionary biologist

At Oxford, de Beer was influenced by and worked with three of the leading zoologists of the time, J. B. Haldane, J. S. Huxley and E. S. Goodrich. His introduction to experimental embryology included two visits to the laboratory of Hans Spemann, who from 1919 to 1937 was Professor of Zoology at the University of Freiburg. This experience culminated in de Beer writing, with J. S. Huxley, *The elements of experimental embryology* [11], the first book to emphasize the importance and perhaps even to recognize the field of experimental embryology, which came to dominate biology in the 1930s and 1940s in the way molecular biology would in the 1960s. In 1935, Spemann received the Nobel Prize in Physiology or Medicine for his and Hilde Mangold's research on the requirement for embryonic induction to initiate the development of organ systems in vertebrates.

The search for the mechanistic basis of embryonic inductions has successively involved the analysis of tissue (epithelial–mesenchymal) interactions, cell–cell interactions, a search for molecular inducers, and, most recently, the discovery of shared gene-signalling pathways as the bases for the initiation of cell differentiation and organ formation [12–15]. Each phase has been accompanied by new approaches to the identification of the basic units (modules) of development and of morphological evolution (see below).

### (c) Skulls

Paradoxically, after his return from Spemann's laboratory in Germany, and despite having co-authored *The elements of experimental embryology,* de Beer abandoned experimental embryology to study the comparative and descriptive embryology of skulls of all classes of vertebrates. As recognized by developmental biologists [16] and philosophers of science [17] alike, this transition from experimental to comparative embryology laid the foundation for de Beer's lifelong contributions to and renown in the field of development and evolution. de Beer examined large wax and plaster of Paris models of skulls carefully reconstructed from serially sectioned embryos. From his Oxford mentor E. S. Goodrich, de Beer recognized the importance of documenting patterns of morphological evolution, and he did so at a time—the 1930s and 1940s—when embryology generally was not considered as having much to contribute to what would become known as the Modern Synthesis of evolutionary biology. These extensive and laborious studies culminated in his monumental *The development of the vertebrate skull* [18].

## 3. Heterochrony and evolution

In his studies on skulls, and as laid out in *Embryology and evolution* ([19]; revised as *Embryos and ancestors* [20,21]), de Beer stressed the importance to evolution of changes in the timing of developmental events in a descendant species when compared with those in an ancestor, a phenomenon known as heterochrony. He paid particular attention to the retention of juvenile features in adults (paedomorphosis) and to reproduction at a juvenile stage in the life cycle (neoteny). Interestingly, these ideas coincided with those of Sewertzoff [22] in Russia, who was developing his own theory regarding the importance of evolutionary novelty and increased organization/integration (aromorphosis) during morphological change. Many biologists accepted these processes because they provided a mechanistic link between embryology and evolution. de Beer summarized his conclusions concerning morphological evolution as follows:

(i) evolutionary novelties can appear at any stage in ontogeny;
(ii) the time and sequence of appearance of characters during ontogeny can change when compared with the ancestral condition;
(iii) such changes introduce novelties into ontogeny and phylogeny; and
(iv) different characters of an organism can evolve by different means [21, p. 88].

de Beer captured the essence of heterochrony in a BBC broadcast on 19 September 1950: ‘By delaying its processes of development, an animal can, as it were, fail to grow up …. I believe that this Peter-Pan type of delayed development has been of the greatest importance in the evolution of animals' [23, p. 62].

With these important and original contributions to theoretical biology, de Beer should be considered one of the early pioneers of the Modern Synthesis. By the same token, *The elements of experimental embryology* should be regarded as the first book that foreshadowed the Modern Synthesis, especially in terms of giving embryology a prominent place in evolution. Paradoxically, later books that are widely considered to have lain the basis for the Modern Synthesis—Dobzhansky [24], Huxley [25], Mayr [26], Simpson [27] and Stebbins [28]—omit or downplay embryology in favour of population and genetic approaches, emphasizing natural selection and not development as the primary mechanism that initiates evolutionary change.

de Beer's mentor and co-author Julian Huxley made his own fundamental contributions to the study of heterochrony. In analyses of proportional change in size and shape between parts of an organism during growth, he and Georges Teissier independently developed the allometric formula, *Y* = *bx^α^*, and agreed on the term allometry for the expressed relationships ([29,30]; see also [31,32]). Huxley had already thought long and hard about such issues, as summarized in his influential book *Problems of relative growth* [33].

Surprisingly, given the importance de Beer attributed to heterochrony as a mechanism of evolutionary change in ontogeny and phylogeny, he mentions it just twice in *The development of the vertebrate skull* [18]: once to explain the then well-known example—cited extensively by Julian Huxley—of allometric growth between upper and lower jaws in some fishes associated with elongation of the ‘snout' (along with brief comments on facial and skull growth in primates; [18, pp. 471–472]); and secondly, in evaluating how to choose appropriate embryonic stages when attempting phylogenetic comparisons [18, pp. 447–448]. In the former case, de Beer identifies two distinct rates of mandibular growth—an initial rapid rate and a subsequent slower rate—with the changeover from one to the other often coinciding with the onset of ossification, especially in amphibians with a metamorphic life history.

In amphibians, initial formation of most, and in some cases all, cranial ossification centres accompanies other, widespread anatomical changes that constitute metamorphosis from an aquatic to a terrestrial stage—changes that are largely under hormonal control [34]. de Beer discussed which of cartilage or bone formation (chondrogenesis or osteogenesis) is the more reliable to document such changes. In his agenda of special problems related to experimental morphogenesis, he asks ‘Can the time-relations of the appearance of bones be modified? What is the sequence of ossification in (*a*) thyroidectomized frog tadpoles; (*b*) precociously metamorphosed frog or (*c*) axolotl?' [18, p. 515]. The sequence and pattern of cranial ossification in both frogs and salamanders would eventually be shown to be regulated by thyroid hormones (e.g. [35–37]). Moreover, independent response by cranial cartilage and bone to endocrine factors underlies the high level of morphological integration between these two skeletal tissues during metamorphosis [38]. Indeed, the skeleton is now known to function as an endocrine organ through life, and not only in animals with a metamorphic life history [39].

Some four decades after publication of de Beer's treatise on the skull, a resurgence of interest in heterochrony as *the* mechanism linking ontogenetic and phylogenetic change came with Stephen Jay Gould's book *Ontogeny and phylogeny* [31], which reviewed the history of the field and reduced de Beer's eight categories of heterochrony to two—*acceleration* and *retardation* of development. These two changes in developmental rate result, respectively, in *recapitulation* or *paedomorphosis*, the earlier or later appearance of a character in a descendant than in its ancestor. Alberch *et al*. [40] subsequently proposed a more explicit scheme of ontogenetic trajectories to visualize heterochrony. Overall, the re-emergence of heterochrony in the 1970s and 1980s reinvigorated research that sought to link changes in the timing of embryonic development to morphological evolution [41]. Applications to cranial evolution ranged from the origin of the highly derived skull of snakes [42] to modularity and the link between cranial development and brain size in mammals [43].

Katz [44] related the constants of the allometric formula used to compare organ growth in adults, *Y* = *b*x*^α^*, to the relative number of cell division centres (*b*) and the difference between the rates of cell division (*α*) of the two organs (*Y* and *x*) in embryos, thereby providing greater understanding of a relation that de Beer had identified as ‘the manifestation of different rates of histogenetic activity in different regions' [18, p. 448]. Initial size and the timing of formation of cell populations (condensations, modules) were subsequently proposed as the cellular bases for such heterochronic change ([12,45–47]; see §6.b).

Developmental processes mediate the nature and amount of variation exposed to natural selection, in some instances constraining the direction of evolutionary change, in others facilitating the origin of novel morphological arrangements and features. The difficulty in gaining access to critical developmental stages in ancestral species led to comparisons of ontogenetic sequences—including the timing of organ initiation—among related extant species (sequence heterochrony), essentially as foretold by de Beer as quoted above. In one example, after comparing Neanderthal and anatomically modern human skulls, Zollikofer & Ponce de Léon [48] concluded that ‘early ontogenetic modifications of a small set of [cranial] growth parameters is a major source of evolutionary novelty during hominid evolution' [48, p. 322]. Both Gould [31] and Zelditch [49] cite further examples and analyses.

## 4. Homology

Section II (Systematic Section) of *The development of the vertebrate skull* [18, pp. 41–373] reflects de Beer's emphasis on two themes: homology, and refutation of the biogenetic law that ontogeny recapitulates phylogeny (see Gould [31] and Hall [50] for overviews of the latter from different perspectives). de Beer had long been unambiguous about the relationship between ontogeny and phylogeny as articulated in the biogenetic law. In the concluding page of the revised edition of *Embryos and ancestors*, for example, he states ‘Clearly, phylogeny does not explain ontogeny at all …. But since phylogeny is but the result of modified ontogeny, there is the possibility of a causal analytic study of present evolution in an experimental study of the variability and genetics of ontogenetic processes' [21, p. 142].

In his search for basic units (modules) of the skull, de Beer contributed a great deal to our understanding of homology, perhaps most notably that homology can be identified at different levels of biological organization, without requiring a common basis in a germ layer or shared genes. de Beer examined and rejected prevailing criteria to identify homology. He dismissed (a) origin from common germ layers, (b) origin by the same embryonic inductions, and (c) a common genetic basis. Yet, and as discussed below, all three criteria have stood the test of time. de Beer concluded that:

— ‘correspondence between homologous structures cannot be pressed back to similarity of position of the cells of the embryo or the parts of the egg out of which these structures are ultimately differentiated' [7, p. 3]. The origin of the alimentary canal was a prime example.
— ‘homologous structures can owe their origin and stimulus to differentiate to different organizer-induction processes without forfeiting their homology' [7, p. 13]. Requirement for induction in lens formation in congeneric species of frogs was a prime example.
— ‘characters controlled by identical genes are not necessarily homologous …. Therefore, homologous structures need not be controlled by identical genes, and homology of phenotypes does not imply similarity of genotypes' [7, p. 15]. The basis for eye formation in *Drosophila* was a prime example. See Hall [51,52] for detailed discussions.

## 5. Segmentation/modularity

### (a) Of animals

For centuries, documenting the existence, nature and developmental basis of segmentation in myriad types of animals has been an important approach to understanding how bodies are organized [13,53,54]. Segmentation may involve the entire body (annelids), specific regions (arthropods) or individual parts of an organ system, such as the vertebral column and vertebrae (vertebrates). All these arrangements have been subsumed under the term segmentation, although seemingly similar cases of repetition may be fundamentally different, evolutionarily independent and so not directly comparable (see the informative discussions in [55]). In the latter half of the nineteenth century and the first decades of the twentieth, research on the vertebrate head as a series of segments played a fundamental role in discussions of the relationships between vertebrates and invertebrates, of the recognition and reclassification of vertebrates as chordates, and of some ‘invertebrates' as chordates.

### (b) Of skulls

Reflecting its importance overall, de Beer devoted a quarter of the 40-page Introduction of *The development of the vertebrate skull* to segmentation. He explicitly accepted Balfour's claim, based on extensive study of shark skulls [56],^4^ that ‘in early stages of development the head is segmented in a manner precisely similar to the trunk' [18, p. 15] and distinct from segmentation of the visceral (gill) arches. de Beer *did* recognize that any ancestral segmentation of the bones of the skull would have become obscured during vertebrate evolution. For de Beer, the primary issue remaining was to determine, using late-stage embryos, the number of segments that contribute to skull development in individual taxa. Cranial segmentation, he believed, was reflected primarily in the head mesoderm. The *fact* of segmentation was taken as a given.

### (c) Neural crest and mesoderm

Most of the skull and the entire jaw skeleton, as well as the connective tissue associated with head muscles, are derived not from mesoderm but rather from (ectodermal) neural crest cells [57,58]. Indeed, the majority of the skull in all vertebrates is derived from neural crest cells [59]. Because neural crest cells are not present in invertebrates and because of the extensive role played by neural crest cells in head formation, the vertebrate head has been defined, evolutionarily, as a ‘new head' [60].

**Figure 1.**
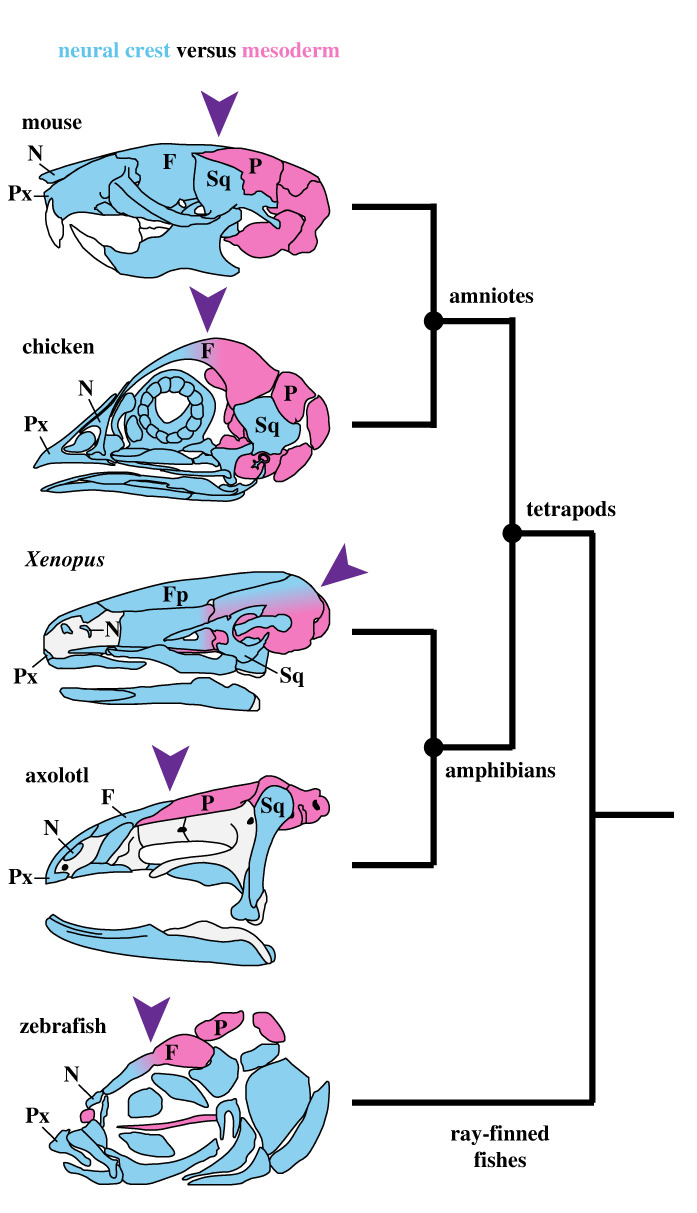
Embryonic origin of the bony skull in five vertebrate model organisms arrayed on a simplified vertebrate phylogeny. Neural crest-derived territories (blue) have been verified experimentally in all five species. Derivation of remaining components from mesoderm (red) has been verified experimentally in mouse and chicken and is presumed for the remaining species. The pattern of embryonic derivation, including the location of the neural crest–mesoderm interface in the skull roof (arrowhead), appears similar in a mammal (mouse), bird (chicken) and urodele (axolotl). It may represent a phylogenetically conserved pattern that is ancestral for all tetrapods. Illustration is redrawn from Piekarski *et al*. [61], which cites data sources. F, frontal; Fp, frontoparietal; N, nasal; P, parietal; Px, premaxilla; Sq, squamosal.

Long-term fate mapping of neural crest cells in the Mexican axolotl (*Ambystoma mexicanum*) and the African clawed toad (*Xenopus laevis*) allowed Piekarski *et al*. [61] to compare the relative contributions of different neural crest streams to bones of the adult skull in these two amphibians. By extending their comparisons to other vertebrates, they were able to demonstrate that the pattern of neural-crest derivation in the axolotl, which essentially is identical to that seen in amniotes, likely represents the ancestral condition for tetrapods (figure 1). A second major conclusion, derived by comparing the unique pattern in *Xenopus* with that shared by other tetrapods, relates to the evolution of developmental processes: ‘interspecific divergence in developmental processes that underlie homologous characters occurs with little or no concomitant change in the adult phenotype' [61, p. 1].

As for the mammalian skull, the development of permanent cell lineage-specific genetic markers in transgenic mice (*Wnt1-Cre/R26R* for neural crest cells*, Wnt1-cre/Mesp1-cre* for mesodermal cells) reveals that rostral skull bones arise from neural crest cells but that more posterior cranial bones (and vertebrae) are mesodermal in origin [62,63]. Indeed, using genetic analysis, the mammalian premaxillary bone has been proposed to be a novelty with a different developmental origin from more posterior skull bones and from the premaxilla of other tetrapods [64]. These results offer convincing insights into the developmental relationship between, and confirm the non-homology of, vertebrae and adjacent elements of the skull [62,63,65,66].

## 6. Skull diversity and evolution

Following the analytical paradigm established by de Beer [18], a major three-volume analysis of the skull edited by Hanken & Hall [67–69] was organized around developmental mechanisms (vol. 1), structural and systematic diversity (vol. 2) and evolutionary mechanisms (vol. 3). We also contributed a summary highlighting important mechanisms of skull diversity and evolution of contemporary interest [70]. These themes have been emphasized to varying degrees in subsequent studies, reflecting the appropriateness of particular taxa for examining certain problems and, in some cases, their suitability for experimental manipulation.

### (a) Vertebrate phylogeny

Although skull development can serve as a source of data for phylogenetic analysis, de Beer was reluctant to draw phylogenetic inferences from the data he amassed, emphasizing instead that future work and especially additional sources and types of data were required. Many fundamental aspects of vertebrate phylogeny unresolved in 1937 have now been resolved. Examples cited by de Beer include the relationships between cyclostomes and fossil jawless fishes, whether birds arose from reptiles, whether lobe-finned fishes are the closest tetrapod ancestors, and the relationships among the three orders of modern amphibians.

de Beer was keenly aware of the importance and power of experimental studies; he had formal training as an experimental embryologist, although the bulk of his personal research lay primarily in static descriptions. He devoted the last chapter of his ‘big skull book' to ‘Causal relationships in the development of the skull,' noting in the preface that ‘a glance at my chapter … will show how meagre is the information in this field' [18, p. xxx]. We will but skim the surface of such work with discussions of three topics: the origin of mammals from reptiles, the evolution of middle ear ossicles from lower jaw cartilages, and the modularity of the mammalian dentary.

### (b) The transition to mammals

Analysis of skull development in a wide variety of recent mammals carried out in the 1970s challenged several long-held views of the so-called reptile–mammal transition and the radiation of modern mammals [71]. Mammals are now known to have arisen from cynodonts. The first cranial evidence of this transition is the transformation of the quadrate and articular bones—which form the jaw articulation in non-mammalian tetrapods, including reptiles—into the incus and malleus, two of the three ossicles of the mammalian middle ear [72,73]. de Beer would have regarded the reptilian origin of mammals as a singular evolutionary event. He never could have anticipated that selection for a more active terrestrial lifestyle would be evidenced in multiple origins of ‘mammalness’ (see Julian Benoit [74] in this issue). Indeed, the middle ear itself evolved independently at least four times in early terrestrial tetrapods [75,76].

Developmental and palaeontological analyses provide the basis for our current understanding of this transition. Developmentally, early in ontogeny of the grey short-tailed opossum, *Monodelphis domestica*: (i) the mandibular arch develops more rapidly than the proximal parts of the hyoid arch, such as the stapes (heterochrony); (ii) phylogenetically older skeletal elements develop earlier than phylogenetically younger elements; and (iii) neonates have neither a typical mammalian nor a typical reptilian jaw articulation (plasticity) [72,77,78]. Palaeontologically, in several Mesozoic mammals on the stem to extant placentals and marsupials, such as *Yanoconodon allini* (a triconodont) and *Maotherium sinensis* (a symmetrodont), the ear ossicles and lower jaw are connected by an ossified Meckel's cartilage. This represents a transitional stage in the origin of ear ossicles from bones of the mandibular arch, which occurred independently three times in the evolution of crown mammals [79–82]. Mechanistically, ossification of Meckel's cartilage in these extinct species allows the direct connection between ossicles and the lower jaw to be retained, a condition otherwise seen only in embryos of extant marsupials and a likely instance of paedomorphosis, a kind of heterochrony [83]. Discovery of an ossified Meckel's cartilage in both extinct therian clades (triconodonts and symmetrodonts) is unexpected from the comparative embryological framework set out by de Beer (and by his mentor, E. S. Goodrich), which dominated discussion of the evolution of middle ear ossicles for many decades before the 2000s.

We now know that, in extant mouse embryos, a single chondrogenic condensation for the lower jaw segregates into (i) the rostral symphyseal cartilages, (ii) a fibrous ligament that replaces most of the rod-like condensation, and (iii) a proximal component that lies at the boundary between the first (mandibular) and second (hyoid) arches and from which the proximal part of the mandibular (Meckel's) cartilage and the malleus arise [47,84]. The latter component provides a developmental basis for connections between the mandibular cartilage and middle ear ossicles and, along with the palaeontological data, affords vital context for the interpretation of molecular studies (see below).

In addition to embryos and fossils, a third class of evidence reinforces phylogenetic inferences. The evidence is molecular—specifically, expression patterns of homeobox (*Hox*) and growth-factor genes in mice and the interpretation of gene-knockout experiments that result in loss of function. Both the mandibular cartilage *and* middle ear ossicles are duplicated in mice in which the homeobox gene *Hoxa2* is knocked out, a treatment that induces a homeotic transformation of the second arch to a duplicate first arch [85,86].

Strong corroboration between developmental and palaeontological evidence is seen in mice in which the gene for Transforming growth factor β2 (Tgf-β2) is knocked out: mandibular cartilage undergoes ossification, and the ossified cartilage is structurally similar to the ossified mandibular cartilage in Mesozoic mammals (reviewed in [82]). DiFrisco & Wagner ([15], and references therein) present the latest gene-based mechanistic model of body plan evolution based on such results, while Kourki [87] discusses the implications of such models for our understanding of the developmental–genetic basis of homology.

Using development to inform phylogeny must take into account variation at different levels of the taxonomic hierarchy [71,88,89]. Furthermore, and as introduced earlier, development itself evolves, especially as a basis for adaptive evolution of embryonic, larval and adult stages of the life cycle. For example, in some viviparous caecilians—elongate, limbless amphibians that make up the order Gymnophiona—the pattern of cranial ossification relates almost entirely to specialization for fetal maintenance via maternal oviducal secretions and provides little information of use for inferring higher-order phylogenetic relationships [90]. On the other hand, development of middle ear ossicles provides an abundance of phylogenetic information at the level of the reptilian–mammalian transition.

### (c) The mammalian dentary

The mammalian lower jaw, also known as the dentary or mandible, is an extraordinary example of modular organization based on multiple cell populations. While comprising a single bone in adults, the dentary actually is a composite bone composed of the ramus (body), its largest component, which ossifies as a membrane bone, and three posterior processes (condylar, coronoid and angular) that develop both by intramembranous ossification as posterior extensions of the ramus and by endochondral ossification of cartilage that caps each process. Bone associated with the teeth (alveolar bone) also contributes to the dentary. Indeed, in rodents, much of the ramus is occupied by the extended roots of the incisor teeth and associated alveolar bone [46,47,84].

Each of these modules is a developmental, functional and evolutionary unit, with their origins in separate populations of cells and responsive to different local environmental factors [46,47,84]. For example, development of the ramus is primarily affected by the teeth, especially in rodents. By contrast, development of the posterior processes is primarily affected by the action of muscles that close the jaw. Individual muscles insert onto individual processes—the temporal muscle onto the coronoid process, the masseter muscle onto the condylar—and failure of an individual muscle to form or experimental inactivation of a muscle set only affects the growth of its corresponding posterior process [46,84]. Consequently, the three processes can vary independently, as seen repeatedly in mammalian evolution.

Furthermore, development and growth of the three processes is mediated by different gene networks. In mice in which the protein-coding gene *Msx1* is knocked out, teeth and the alveolar bone that normally supports the teeth fail to form but the three posterior processes develop normally. Conversely, gene knockouts for the homeobox protein Goosecoid or for growth factor Tgf-β2 show reduced growth of all three processes but no effect on teeth or alveolar bone [91]. Such module-specific genetic control provides a mechanistic basis (i) for the independent evolution of individual components of the dentary [84], (ii) for the genetic–developmental basis of modularity, (iii) for homology, and (iv) for the long-term preservation of body plans [15,87].

## 7. Concluding remarks

As the present collection of papers attests, not only has de Beer's research on the mammalian skull influenced the field for the past 75 years, but it continues to inform our approach to the most fundamental questions concerning skull development and evolution across the vertebrates. Whether and how the head is segmented, how and the extent to which neural crest and mesodermal cells (which arise from different germ layers) form different parts of the skull, how cells function as modular units in skeletal development and evolution, how changes in the timing of development can mediate large-scale skeletal changes, and the bases on which the homology of skeletal elements can be determined, are major themes. All have their roots in de Beer's research.

## Data Availability

This article has no additional data.

## Endnotes

1 Commonly known as fishes, amphibians, reptiles, birds and mammals but comprising seven taxonomic classes; https://www2.palomar.edu/anthro/animal/animal_4.htm.

2 Details of de Beer's life may be found in two major biographical sources, Barrington [8] and the entry under ‘Gavin Rylands de Beer’ in *Hutchinson dictionary of scientific biography* [9].

3 Women scientists at the museum were few in number. None received a full salary, usually being ‘paid’ by the piece for specimens they collected or prepared, but they made outstanding contributions in diverse fields. The accomplished Beatrix Potter (writer, illustrator, natural scientist and conservationist) illustrated flora, Dorothea Bate (explorer and palaeontologist) collected mammal fossils from the Mediterranean, Lucy Evelyn Cheesman explored and collected insects in the South Pacific, Sidnie Manton was a world expert on arthropods, and Juliet Clutton-Brock, who was a full-time member of the museum from 1969 to 1993, was a world authority on the domestication of animals [10, pp. 269–275].

4 Published initially as a series of seven papers between 1876 and 1878, these papers were subsequently collected by Balfour into a four-volume monograph on the development of elasmobranch fishes [56].
